# High-resolution cryo-electron microscopy on macromolecular complexes and cell organelles

**DOI:** 10.1007/s00709-013-0600-1

**Published:** 2014-01-05

**Authors:** Andreas Hoenger

**Affiliations:** Department of Molecular, Cellular and Developmental Biology, University of Colorado at Boulder, Boulder, CO 80309 USA

**Keywords:** Cryo-electron microscopy, Cryo-electron tomography, 3-D reconstruction of macromolecular complexes, CTF-correction, Volume-averaging, 3-D particle classification

## Abstract

Cryo-electron microscopy techniques and computational 3-D reconstruction of macromolecular assemblies are tightly linked tools in modern structural biology. This symbiosis has produced vast amounts of detailed information on the structure and function of biological macromolecules. Typically, one of two fundamentally different strategies is used depending on the specimens and their environment. A: 3-D reconstruction based on repetitive and structurally identical unit cells that allow for averaging, and B: tomographic 3-D reconstructions where tilt-series between approximately ±60 and ±70° at small angular increments are collected from highly complex and flexible structures that are beyond averaging procedures, at least during the first round of 3-D reconstruction. Strategies of group A are averaging-based procedures and collect large number of 2-D projections at different angles that are computationally aligned, averaged together, and back-projected in 3-D space to reach a most complete 3-D dataset with high resolution, today often down to atomic detail. Evidently, success relies on structurally repetitive particles and an aligning procedure that unambiguously determines the angular relationship of all 2-D projections with respect to each other. The alignment procedure of small particles may rely on their packing into a regular array such as a 2-D crystal, an icosahedral (viral) particle, or a helical assembly. Critically important for cryo-methods, each particle will only be exposed once to the electron beam, making these procedures optimal for highest-resolution studies where beam-induced damage is a significant concern. In contrast, tomographic 3-D reconstruction procedures (group B) do not rely on averaging, but collect an entire dataset from the very same structure of interest. Data acquisition requires collecting a large series of tilted projections at angular increments of 1–2° or less and a tilt range of ±60° or more. Accordingly, tomographic data collection exposes its specimens to a large electron dose, which is particularly problematic for frozen-hydrated samples. Currently, cryo-electron tomography is a rapidly emerging technology, on one end driven by the newest developments of hardware such as super-stabile microscopy stages as well as the latest generation of direct electron detectors and cameras. On the other end, success also strongly depends on new software developments on all kinds of fronts such as tilt-series alignment and back-projection procedures that are all adapted to the very low-dose and therefore very noisy primary data. Here, we will review the status quo of cryo-electron microscopy and discuss the future of cellular cryo-electron tomography from data collection to data analysis, CTF-correction of tilt-series, post-tomographic sub-volume averaging, and 3-D particle classification. We will also discuss the pros and cons of plunge freezing of cellular specimens to vitrified sectioning procedures and their suitability for post-tomographic volume averaging despite multiple artifacts that may distort specimens to some degree.

## Background

### The evolution of cryo-electron microscopy

The nature and quality of electron microscopy image data has changed dramatically with the introduction of cryo-electron microscopy (cryo-EM) and specimen vitrification. Cryo-EM became truly popular in the 1980s. Since the pioneering works of structural biologists such as Jacques Dubochet (e.g., see Adrian et al. 1984; Dubochet et al. 1988), Robert Glaser (Taylor and Glaeser 1976), and others, cryo-EM evolved from a highly specialized niche application, employing complicated and sometimes unreliable equipment, to a widely accepted technology that, as of today, produces large amounts of spectacular detailed structural and functional studies on biological macromolecular assemblies to near atomic detail. Cryo-EM relies mostly on the phase contrast produced by the density differences between protein density and the embedding, vitrified buffer. Frozen-hydrated specimens remain unperturbed from staining and/or fixation and maintain atomic detail (Taylor and Glaeser 1976). However, these bright prospects come at a price; vitrified samples produce low contrast, and, unlike chemically fixed, metal-shadowed, or stained preparations, they are very prone to electron beam-induced damage. Nevertheless, propelled by the constantly improving structural data produced by computer-aided, averaging-based 3-D reconstruction methods for macromolecular assemblies such as helical 3-D analysis (DeRosier and Moore 1970), tilt-series reconstructions from 2-D crystalline arrays (Unwin and Henderson 1975), icosahedral viral capsids (Crowther et al. 1970), or single particle (van Heel and Frank 1981; Frank and van Heel 1982) reconstruction methods, electron microscopists soon felt the limitations of the physical resolution limits imposed by negative staining or metal shadowing specimen preparations and demanded molecular fixation methods that enabled atomic details to be obtained. Atomic detail had been first achieved for macromolecular assemblies on 2-D crystalline arrays, and most recently even on pure single particle reconstructions. The history of reaching atomic resolution reads roughly as follows: (a) diffraction data of protein crystals: Taylor and Glaeser 1974 (catalase crystals); (b) 2-D crystals of membrane proteins: Henderson et al., 1990 (bacteriorhodopsin; current world record was achieved with acquaporin-0 at 1.9 Å (Gonen et al. 2005); (c) 2-D crystalline arrays of soluble proteins: Nogales et al. 1998 (tubulin); (d) helical assemblies: Miyazawa et al. 2003 (acetylcholine receptor reconstituted in lipid tubes) and Yonekura et al. 2003 (bacterial flagella); (e) icosahedral particles: Liu et al. 2010 (adenovirus); (f) single-particle reconstructions: Li et al. 2013 (proteasome).

### From noisy cryo-EM 2-D projections to atomic-detail 3-D reconstructions

Averaging over hundreds and thousands of identical image elements is an incredibly powerful method to reduce electron microscopy-based image noise, by now often far enough to reveal atomic resolution structural detail. Obviously, averaging can only succeed on structurally identical particles that can be precisely aligned according to their various angular orientations in the recorded 2-D projections. Where applicable, structural variations such as ligand-induced conformational changes have to be recognized and classified accordingly. It is therefore no surprise that 2-D crystals with their strict particle packing regime were among the first macromolecular assemblies from which near-atomic 3-D structural detail was obtained (Taylor and Glaeser 1976; Henderson et al. 1990), while single particle reconstructions only now, and with the help of the latest technology developments on software and hardware (e.g., direct detectors Li et al. 2013; Veesler et al. 2013), seem to promote the breakthrough to near-atomic detail. Thanks to the regular packing of individual units into a highly repetitive structure, with its very practical consequences for Fourier space aided separate analysis of phases and amplitudes, 2-D crystals, helices, and to some extent also icosahedral particles enable the analysis of assemblies of very small (<40 kDa) unit cells. Already the packing procedure into a crystal or a helix acts as a physical filter that either only truly admits identical units, or otherwise provides poor crystallographic packing, which will render these assemblies useless for high-resolution analysis (e.g., see Kühlbrandt 1992; Hite et al. 2007). Solubilized single particles such as ribosomes, proteasomes etc. do not assemble into any type of regular array. Hence, electron micrographs typically present them as sets of 2-D projections along very different directions. The most even distribution of projection angles warrants isotropic resolution without a need for recoding tilts. Nevertheless, the signal/noise ratio has to be sufficient to unambiguously reveal the angular relationships between particles, provided they are all identical. If this is not the case, classification has to succeed in identifying distinctly different conformational states (van Heel and Frank 1981; Frank and van Heel 1982; Frank 2009).

### 3-D cryo-electron microscopy on large, flexible structures

**Fig. 1:**
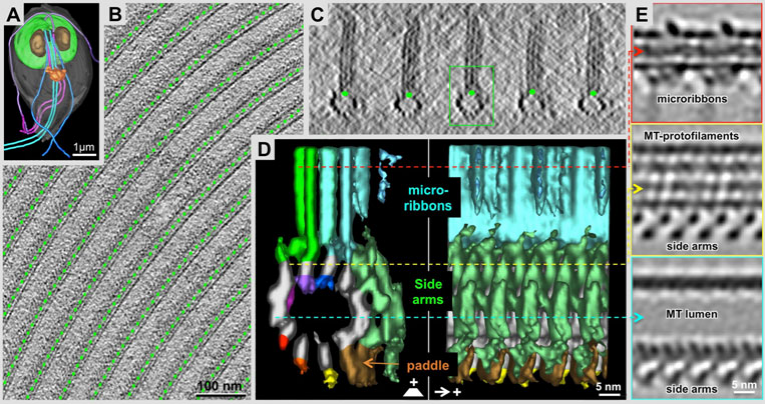
Subvolume averaging from tomograms of plunge-frozen, regular arrays within the unique *Giardia lamblia* cytoskeleton. **a** Microtome-based block-face scanning-EM imaging of a *Giardia* trophozoite reveals sufficient resolution to identify flagella, nuclei (*N*), the median body (*MB*), and ventral disc (*VD*). These arrays constitute excellent test grounds for our labeling experiments as they allow averaging of subvolume elements and calculating difference maps for a precise detection of clonable labels. **a** Tomographic 20-nm thick X-Y slice of the *Giardia* ventral disc (green organelle in the *Giardia* cell shown in (**c**, **d**) at the level of the microtubule arrays. **c** Tomographic 20-nm thick X-Z slice of the *Giardia* ventral disc showing microtubules and associated microribbons end-on. **d**, **e** 3-D reconstruction of the microtubule-microribbon complex of the Giardia ventral disc. **d** End-on view (*left panel* towards the microtubule plus-end) and side view (*right panel*) of a grand average over 4,700 individual tomographic subvolumes. MT protofilaments are numbered, starting at the position of the seam. The largest associated densities are called side-arms (*green*). Currently, we do not know how many individual protein domains are within this structure. **e** *Panels top* to *bottom* show cross-sections at corresponding position in the 3-D map marked by lines of the following color: *red* (microribbons), *yellow* (upper microtubule protofilaments), and *cyan* (microtubule lumen and side arm densities). The cross-section through the microribbons reveals a distinct 16-nm repeat, corresponding to two consecutive αβ − tubulin dimers along a protofilament. Side-arms repeat in register with the tubulin dimer repeat. The 3-D map still suffers from a missing cone of data (see also Fig. 4), demonstrated by the clear separation of protofilaments horizontally, but not vertically (for further details see Schwartz et al. 2012)

**Fig. 2:**
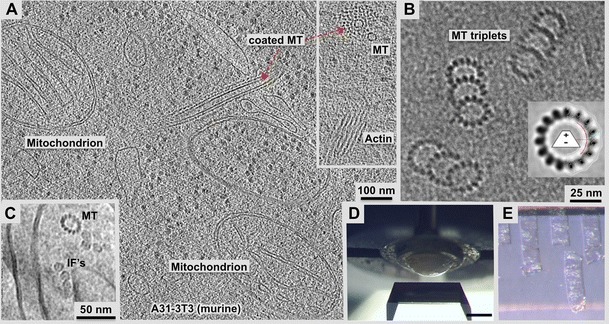
3-D analysis of large cells and tissues: Intracellular molecular details preserved in vitrified sections. **a**, **b** 70 nm, frozen-hydrated cryo-section observed in a high-pressure frozen A31-3 T3 cell. **a** Approximately 10-nm thick tomographic slice that shows mitochondria, ribosomes, endosomes, and an actin-coated microtubule. Inset: end-on view of such a coated microtubule, and side view of an actin bundle. **b** An important advantage of CEMOVIS is illustrated in these triplet microtubules of the centriole where the technique allows for a direct determination of the microtubule polarity due to the characteristic slew of the protofilaments in end-on views (compare to helical averaging of a set of microtubules in the inset). Here, we are looking from the minus towards the plus end. **c** Cross-sections of a microtubule next to vimentin intermediate filaments (*IFs*) in a vitrified section of CHO cells. All of the vimentin filaments show a clear central density, which is different from data obtained on in vitro polymerized vimentin IFs (see Goldie et al. 2007). **d** Frozen cell pellet in a dome-shaped carrier approaching the cryo-diamond knife for trimming, **e** 100-μm wide, 50-nm thick vitrified sections are shown on the surface of a 45° cutting cryo-diamond knife ready to be collected on a carbon-coated, copper grid. Cryo-sections have a transparent to silver, shiny look. Unlike plastic sections, vitrified sections cannot be floated on a water surface for obvious reasons. Hence, they have to be picked up by a micromanipulator and transferred to an EM grid

**Fig. 3:**
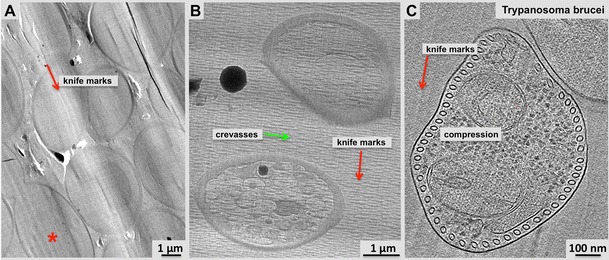
The vitrified section process affects the specimen geometry in various ways: Damage by the cutting knife such as compression or crevasses and its effect to macromolecular structures has to be carefully analyzed before volume averaging procedures can be applied. Large-scale distortions and compression along the cutting directions (*red arrows*) affect membrane organelles, including microtubules cross-section and shape (MT in **a**, **c**). **a** 2-D projection micrograph through a vitrified section of 3T3 cell showing multiple membrane vesicles and some microtubules towards the center. Most organelles appear compressed along the cutting direction. **b** 2-D projection micrograph of a vitrified section through *S. pombe* cells revealing the typical distortions encountered during vitrified sectioning such as knife marks and crevasses. **c** 21-nm tomographic slice through a cross-section of a *Trypanosoma brucei* cell reveals the spectacular microtubule-based cytoskeleton of this single-cell organism around the edge. Most of the microtubules appear squeezed roughly along the direction of the knife marks, though the compression is not uniform (see also Höög et al. 2012)

The spectacular success of 3-D reconstructions by cryo-EM and averaging procedures should not make us forget the fact that the 3-D architecture of the vast majority of biological structures cannot be subjected to averaging methods. The larger a macromolecular assembly or cell organelle is, the higher the probability of intrinsic flexibility, and the lower are the chances of structural identity between multiple copies (e.g., Figs. 1, 2, and 3). If averaging and/or alignment procedures of particles fail, the only way to obtain 3-D data is cryo-electron tomography (cryo-ET; Medalia et al. 2002; Beck et al. 2004, reviewed in Steven and Aebi 2003; Lucic et al. 2005). Today, high-resolution cryo-ET is probably the most active and fastest emerging discipline in structural cell biology as it can be applied to large macromolecular assemblies, cellular organelles, or even entire tissues. By omitting the need for any kind of averaging procedures, at least during the initial round of 3-D reconstruction, tomography can be applied to any specimen that is thin enough to get an electron beam through. Intact bacterial cells such as *Caulobacter crescentus* (e.g., see Briegel et al. 2006, 2009; multiple examples reviewed in Gan and Jensen 2012), or flat areas of eukaryotic cells (e.g., fibroblast peripheries; Dictyostelium: Medalia et al. 2002) may be suitable for direct imaging. All other cellular specimens have to be treated by vitrified sectioning (e.g., see McDowall et al. 1983; Al-Amoudi et al. 2004a; Dubochet et al. 2007; Bouchet-Marquis and Hoenger 2011), or by focused ion-beam milling in a dual-beam cryo-scanning electron microscope (reviewed in Lucic et al. 2013).

Tilt-series data collection and data processing in electron tomography is much more demanding with respect to computing power and data storage than diffraction-based averaging methods where 2-D and 3-D datasets often can be reduced to rather small sets of numerical structure factors. Individual tomograms (conventional or cryo-ET) easily exceed a gigabyte (GB) in size, and computationally combined super-montages (Mastronarde et al. 2008; currently still a domain of plastic-section tomography) may be as large as 10–20 GB and beyond. Compared to conventional plastic section tomography where grids are often pre-exposed to the beam to avoid shrinking during data collection, cryo-ET adds a complication to the process due to its dramatic sensitivity to the electron beam. The total dose specimens are exposed to during tilt-series acquisition has to be carefully considered and kept below values that would destroy projections towards the end of a tilt series. The resulting individual low-dose images are extremely noisy and may often lack the so-called fiducial markers due to the difficulties when handling vitrified specimens that may complicate the proper alignment of individual tilted projections for the 3-D back-projection process. Recent progress in microscope stage designs as well as camera hardware (direct electron detectors; Jin et al. 2008; McMullan et al. 2009a; Campbell et al. 2012) will be very beneficial for progress in cryo-ET and will be discussed below.

## Exploiting the full potential of cryo-ET 3-D analysis: processing the raw data

**Fig. 4:**
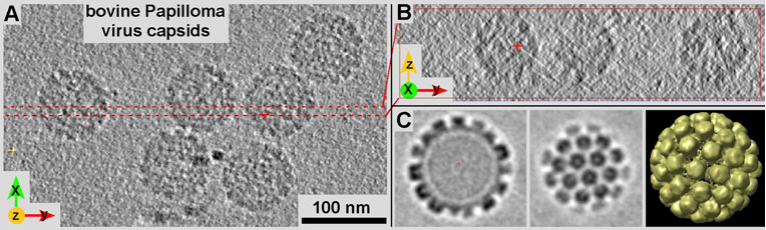
Effects of the missing wedge on the image quality in tomograms illustrated on bovine Papilloma virus capsids. Tomograms are unisotropically resolved, lacking resolution along the *z*-axis (in the case of a missing cone) and *z*-axis and along the tilt-axis (missing wedge). While the XY-view (**a**) typically look highly resolved, the anisotropy of image resolution in tomograms becomes very obvious when viewed along the *x* or *y*-axis (**b**). Typically, the *z*-axis becomes stretched and has less resolution. Averaging multiple, structurally identical particles viewed under various angles may resolve that problem and restore the resolution equally in all directions. **c** 3-D structure of BVP at ~2.5-nm resolution obtained by volume averaging from the data shown in **a** and **b**. After averaging together particles with different orientations in the tomogram, the resolution is now isotropic

Knowing and understanding the challenges that are facing us is typically the first step to success. Sophisticated technologies such as cryo-ET always come with their unique sets of issues and demands. TEM-based cryo-electron tomography of frozen-hydrated samples features several challenges for data acquisition, data analysis, and data interpretation that all require careful consideration. Regarding imaging and data acquisition, frozen-hydrated specimens—due to low intrinsic contrast—are recorded at large underfocus values, typically between −2 and −6 μm, which creates images, mostly from phase contrast that are strongly affected by the contrast transfer function (CTF). The CTF is particularly demanding to assess on highly tilted specimens where focus values gradually vary perpendicular to the tilt axis. In addition, cryo-holders and the geometrical nature of a TEM specimen stage limit the range for tilt series to maximally ±70°, which creates a missing wedge of data (see Figs. 1 and 4). With a maximum tilt of ±60°, the missing wedge reduces resolution along the projection direction and tilt axis by 50 % (Fig. 4b) and affects further 3-D analysis procedures such as alignments (in particular for fiducial-free alignment; Winkler and Taylor 2006; Castano-Diez et al. 2007) and classification of sub-tomogram volumes picked for averaging (Förster et al. 2008; Winkler et al. 2009; Heumann et al. 2011; Yu and Frangakis 2011). However, these kinds of distortions apply to any kind of cryo-sample and each element in a cryo-micrograph or cryo-tomogram. Most of them are quite well understood and several correction procedures have been published from our lab (Xiong et al. 2009) and others (Zanetti et al. 2009; Eibauer et al.,^2012^). However, there is still substantial room for improvement. For cryo-ET, features such as CTF correction (Xiong et al. 2009), and a correction for beam tilt (Mastronarde 2008), are routinely applied.

### Fiducial-less alignments of tilted projections is currently the standard for cryo-ET

Whenever possible, tilt series are aligned to each other with the help of electron-dense fiducial markers, typically gold particles from 5–15 nm in diameter that can be easily added to grids and plastic-thin sections that are handled at ambient conditions (room temperature, no vacuum; Mastronarde 2007). In contrast, fiducial markers are often difficult to incorporate into frozen-hydrated samples (see Masich et al. 2006; Gruska et al. 2008). The *Giardia lamblia* ventral disc structure presented in Fig. 1c, as well as the tomograms of Figs. 2a and 3c, are all products of fiducial-less alignments. Hence, successful alignment procedures for tilt series without fiducial markers are very important for most kinds of plunge-frozen specimens and in particular for vitrified sections. Among the first to tackle this problem was the group of Winkler and Taylor (2006), but their method that relied on tracking image features through the entire set of tilted projections had some limitations. Much greater success was achieved with a novel approach developed by Castano-Diez et al. (2007, 2010): correlating multiple overlapping patches of image through the tilt series and treating the tracked positions like a fiducial model. Because alignment data are available at multiple positions, it is possible to solve for the tilt axis angle, for specimen shrinkage, and even for refined tilt angles in some cases. However, since image patches rather than specific features are tracked, the tracking may not follow a consistent position through the whole series, just as an overall cross-correlation may not. The effects of this positional variation can be ameliorated by subdividing each track into overlapping segments (Castano-Diez et al. 2010). This method has been made available in our own IMOD software package and eTomo interface and is now used routinely. It can be successfully used with vitrified sections, where relatively few large patches are tracked, as well as with stained plastic sections, where many smaller patches can be tracked.

## Exploiting the full potential of cryo-ET 3-D analysis: volume averaging of tomographic data

### High-resolution cryo-ET

**Fig. 5:**
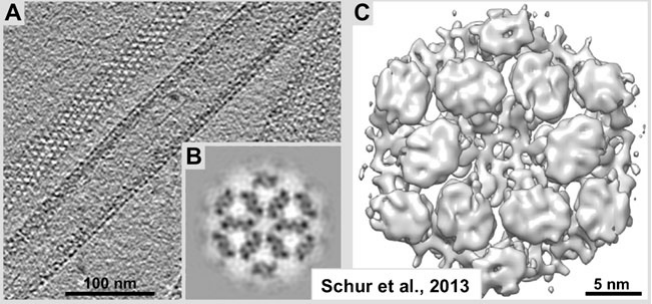
Sub-tomogram averaging reconstruction from tubes of M-PMV (Mason–Pfizer monkey virus) DPro CANC protein (shown in **a**). **b** Gray-level, and **c** iso-surface representations hexameric DPro CANC protein unit cells. For details see Schur et al. 2013. Currently, this reconstruction is among the best-resolved datasets obtained by sub-tomogram averaging. At 8.5 Å resolution, this 3-D dataset from the lab of John Briggs at the EMBL-Heidelberg exceeds all previous expectations and opens the avenue for secondary element studies by cryo-ET. Adapted from Schur et al. 2013, Figs. 2a and 4a, b, with permission from Drs. J. Briggs and F. Schur

We like to speak of “high-resolution cryo-ET.” However, compared to the typical averaging-based methods described above, the meaning of “high” has to be seen in relative terms; for averaging-based 3-D reconstructions today, high-resolution means near-atomic detail. High-resolution cryo-ET datasets today, after producing 2–3-nm detail for some time, now start to break through the 2-nm resolution barrier (<1 nm in some cases; see Fig. 5 and Schur et al. 2013). Beyond 2–3-nm resolution, unambiguously interpretable structures are typically only resolved by post-tomographic volume averaging (e.g., see Nicastro et al. 2006; Cope et al. 2010; Schwartz et al. 2012; Kuybeda et al. 2013; Schur et al. 2013). All other cryo-tomography reconstruction data that have not been averaged in any way, unambiguously interpretable structural data remains in a 3–4-nm range.

### Post-tomographic sub-volume averaging fully exploits the resolution of cryo-ET

Having praised conventional and cryo-ET for their independence from averaging procedures does not mean there is no post-tomographic averaging possible with tomographic 3-D data. Once a tomogram is calculated, there may be numerous objects in such a 3-D dataset that can be averaged in forms of 3-D sub-volumes, provided there are identical particles to be found (e.g., ribosomes; Pierson et al. 2011; Figs. 1 and 4). Aligning 3-D volumes along all three axes increases the demand for fast computers and algorithms, but features substantial advantages over picking and classifying single particles as 2-D projections. As for 2-D projections, averaging structurally identical 3-D volumes improves the signal/noise ratio in these datasets, in an ideal world by about the square root of numbers of particles. However, there is a crucial difference between averaging and 3-D reconstructing 2-D projections and averaging readily available 3-D datasets. Their alignment is computationally much more demanding but reconstructed 3-D volumes already contain much more information than 2-D projections where superimposed densities require careful de-convolution. Sub-volume averaging not only improves resolution but it may be a way to overcome intrinsic tomography-related issues such as filling in missing wedge or cone data by averaging over particles in different orientation (Fig. 1) or with high internal symmetry (Fig. 4).

### Classification and variance maps

As for the regular averaging process described above, 3-D volumes significantly facilitate the alignment process with the benefit of reducing the noise level of cryo-ET data, but also allowing for assessing possible heterogeneity of the particles being averaged and for clustering them into separate classes according to distinct conformational states and/or composition with multiple components (Förster et al. 2008; Heumann et al. 2011; Frank et al. 2012; recently reviewed in Briggs 2013). However, the intrinsic missing tomographic data caused by missing wedges or cones, as well as low signal-to-noise ratio, make classification problematic. In our lab, we have developed a new method for clustering in the face of these difficulties that aims at estimating the difference between observed and expected particles, taking the potential effects of missing wedge data into account. Differences between expected and observed sub-volumes, which we term “wedge-masked differences” (WMDs), can then be analyzed using standard statistical methods (Van Heel and Frank 1981; Frank and van Heel 1982). We developed programs using WMDs for computation of a corrected variance map and for classification based on principal components analysis followed by K-means clustering (Heumann et al. 2011).

Several labs, including our own, have invested substantial effort into the development of volume averaging procedures (e.g., our PEET software package: applications in Nicastro et al. 2006; Cope et al. 2010; Schwartz et al. 2012), classification protocols (Heumann et al. 2011; Frank et al. 2012), as well as corrections for the contrast transfer function (Xiong et al. 2009). Today, volume averaging and classification of 3-D particles is a hot topic in the cellular tomography community (e.g., see Kuybeda et al. 2013; Frank et al. 2012; Yu and Frangakis 2011; Kudryashev et al. 2012; Bartesaghi et al. 2012) and our lab and others continue to be at the forefront of this developing technology.

## Exploring the 3-D structure of intact cells by cryo-ET

### Cryo-electron tomography of vitrified sections

Until recently, cellular EM was synonymous with rapid-freezing and freeze-substitution fixation (RF-FSF) protocols followed by plastic embedding for many biologists (Carlemalm et al. 1985; Kellenberger 1991; McIntosh et al. 2005). In fact, RF-FSF is still the method of choice for investigations into the cellular architecture and the organization of organelles in the cytosol (Figs. 2 and 3; Marsh et al. 2004; Pelletier et al. 2006; O’Toole et al. 2007; Ferguson et al. 2007; Höög et al. 2007; see also article by Kent McDonald, this issue). Like the “pure” cryo-methods, RF-FSF employs an initial vitrification step with a plunge or high-pressure freezer, and as such, it constitutes a reliable method for observing cellular data to about 5-nm resolution (Studer et al. 1989; McDonald and Morphew 1993).

The protocols for vitrified sectioning omit the freeze-substitution and plastic-embedding step and cut sections straight from a block of a frozen-hydrated specimen. Today, the procedure is often referred to as cryo-EM on vitrified sections (CEMOVIS; Al-Amoudi et al. 2004a), and has actually been around for some time (Christensen 1971; McDowall et al. 1983; Hsieh et al. 2002; Al-Amoudi et al. 2004a, b; Dubochet et al. 2007) so that this acronym seems to be part of a Swiss insider joke. Although the first vitrified sections were produced as early as 1971 (Christensen 1971), cryo-electron tomography on vitrified sectioning was significantly facilitated only after substantial technical developments on cryo-EM-related tools such as cryo-holders, plunge-freezers, high-pressure freezers, ultra-sensitive detectors, and last but not least by the availability of fast, efficient computers and software became available (e.g., see Patwardhan et al. 2012). Even today, the technology requires a lot of manual skills and is only used by few labs. Vitrified sectioning reveals images of cellular organelles and macromolecular assemblies in a frozen-hydrated state, thereby omitting both contrast-enhancing staining solutions as well as chemical fixatives such as glutaraldehyde. The most important advantage resulting from cryo-immobilization of biological samples is a superb structural preservation under most native buffer conditions, which closely resembles the living state of a cell and maintains the structure of macromolecules down to atomic detail (Dubochet et al. 1988; Baumeister and Steven 2000; McIntosh 2001; Steven and Aebi 2003). Accordingly, the expectations for structural preservation and achievable image resolution are high, but a realistic assessment of the method's advantages and limitations has to be done before investing in that technology (e.g., see Dubochet et al. 2007; Pierson et al. 2010; Bouchet-Marquis and Hoenger 2011).

Vitrified sectioning, if exploited for its true potential does not compete with, but is very complementary to plastic-embedding and conventional ET. CEMOVIS clearly has the capacity to deliver a different type of information such as more detail from intracellular molecular structures rather than large overviews. Thus, the most important aspect with regard to vitrified sections is the full exploitation of the image resolution since molecular structures are at no time exposed to staining solutions or chemical fixatives. Combined cryo-electron tomography and single-particle 3-D reconstruction protocols (Kuybeda et al. 2013), or sub-volume averaging procedures (Schur et al. 2013), may provide sub-nanometer detail. However, the question remains as to how good are these protocols when transferred to cellular specimens that have been subjected to vitrified sectioning. On a first inspection, the structural information that is directly interpretable in vitrified sections may not be that much richer in detail than from plastic-embedded sections, although this may vary with the object. Typically, the full potential of vitrified sections can only be exploited by additional averaging-based processing methods (see Figs. 1 and 4). A plain 2-D image of a vitreous section that has been recorded under low-dose conditions (~1–5 electrons per Å^2^) may theoretically hold near atomic detail, but low contrast and the super-positioning of cellular densities in a 50–80-nm thick section through dense cellular material makes it difficult to analyze small structures. Furthermore, a cryo-tomogram of a vitrified section is a composite of multiple projections from a tilt series (typically 100–140 at 1–2° intervals) where the final resolution in the tomogram is very dependent on the successful reconstruction and the stability of the specimen in the electron beam. Even in near-perfect tomograms, the signal/noise ratio is still rather low (Fig. 5).

### Compression, crevasses, and other problems

Currently, probably the toughest challenge on vitrified sections comes from distortions that are implemented during the sectioning process (Fig. 3; see Al-Amoudi et al. 2005; Dubochet et al. 2007; Han et al. 2008; Bouchet-Marquis and Hoenger 2011). Vitrified sections are cut through relatively brittle ice, leaving knife marks (Fig. 3a–c) and crevasses (Fig. 3b), particularly at the side facing away from the knife surface. Other common sectioning-induced distortions are compressions (Fig. 3c) that affect different structures in a section with very different severities. However, despite these conditions, vitrified sections preserve high-resolution structures very well (Sader et al. 2009). With regard to exploiting high-resolution data from vitrified sections, it is these compressions in particular that require more careful study. The remaining question is how much is volume averaging of macro-molecular structures affected by compressions, and which kind of molecular structures can still be analyzed by averaging procedures.

## Clonable high-electron dense labels for vitrified specimens

### How to navigate the complexity of proteinaceous densities in vitrified cellular specimen

This final chapter touches an issue that is not only relevant for cryo-EM or cryo-ET, but concerns all cellular electron microscopy in general. Localizing a particular structure of interest within the crowded environment of a cytosol can be very challenging. Recognizing the components of interest at in vitro condition seems straightforward, but even in vitro labels may be very helpful (e.g., see Cope et al. 2013). Unambiguously, identifying a macromolecular assembly within the crowded environment of the cytosol without any type of labeling can be very difficult, if not impossible. Within intact cells, we may easily be able to identify unambiguously microtubules or ribosomes (e.g., see Figs. 1, 2, and 3), but most other structures remain hidden away by the multitude of dense complexes within the cytosol.

**Fig. 6:**
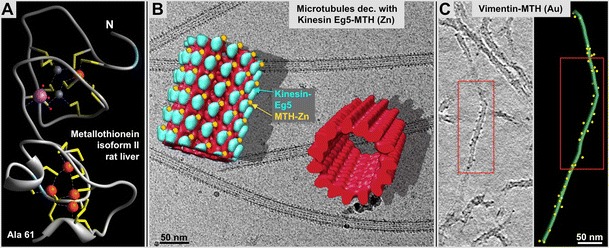
Clonable, gold-clustering labels. Metallothionein (*MTH*) complexed with gold constitutes a clonable high-density marker for electron microscopy (Mercogliano and DeRosier 2006). **a** Atomic-detail NMR structure of rat liver MTH (isoform II) at 2.0-Å resolution (Braun et al. 1992). The structure was solved with five Cadmium ions (*orange*), two Zinc ions (small purple spheres) and one Sodium ion (pink) clustered within the molecule. MTH contains 20 cysteine residues (*yellow* sticks) that achieve the clustering of metal ions by chelation with their cysteine-thiol groups that can accumulate to 20 and more gold atoms within the crystal structure forming easily detectable high-density cluster **b** Metallothionein-Eg5 microtubule complexes with a helically averaged 3-D reconstruction highlighting the MTH-zinc densities (*yellow*) and their position relative to Eg5 (*blue*). A helical average of an undecorated microtubule is shown in *red*. **c** Desmin-MTH chimera polymerized into intermediate filaments present the MTH-gold clusters on their surface. *Left panel* 3-D model of the center filament in C (compare *red frames*). See also Bouchet-Marquis et al. 2012

Plastic-based thin-sections can be handled at room temperature and therefore may be treated with gold-labeled antibody solutions to localize surface-exposed epitopes on sections (e.g., see Tokuyasu 1980). However, the situation is very different with vitrified specimens. The most important differences between a frozen hydrated, or vitrified specimen, and one that is either air-dried or plastic-embedded are as follows: (a) once frozen, a vitrified specimen has to be kept below the devitrification temperature (~133 K, or −140 °C) of water at all times, and (b) cannot be exposed to ambient conditions for more than a few seconds (e.g., during the grid-transfer into the microscope, otherwise the specimen, acting as a cold trap, would be heavily contaminated by ice from humid air. (c) Vitrified specimens are extremely sensitive to the electron beam. That not only calls for expensive equipment such as cryo-holders and high-vacuum stages, but it also prevents essentially every kind of after-freezing treatment with substances that are warmer than 133 K. Hence, there are no protocols for applying antibodies or any other liquids, including solutions with fiducial gold markers, to a vitrified specimen. Hence, all protein tagging has to be done before freezing the specimen, preferably with clonable tags that can be made visible in the electron microscope. Such tags include clonable metal-clustering proteins or peptides (e.g., see metallothionein Fig. 6 and Mercogliano and DeRosier 2006; A3-peptide Slocik et al. 2005), as well as aptamer-based strategies (Stanlis and McIntosh 2003; Feldheim and Eaton 2007), such as linking them to boranephosphate DNA segments or other metal precipitating structures (Roy et al. 2012, 2013).

My own efforts to develop clonable labels dates back to approximately 2002 while I was still working at the EMBL in Heidelberg Germany (Wendt et al. 2002; Skiniotis et al. 2003). Eventually, clonable labels for electron microscopy will fulfill a similar function to clonable labels for fluorescence light microscopy, such as GFP, mCherry, etc. They have the advantage of being directly linked to the protein of interest, providing increased spatial resolution. However, while the tags now work rather well in in vitro situations (Bouchet-Marquis et al. 2012), their applicability within living cells has to be proven first. Clonable labels that are relevant for electron microscopy studies may be placed in three classes as follows: (a) simple protein domains such as an SH3 domain (e.g., see Wendt et al. 2002; Skiniotis et al. 2003), (b) peptides that reduce metal ions into electron dense clusters (e.g., the gold-clustering A3-peptide; Slocik et al. 2005), or (c) protein domains that chelate metal ions with reactive elements such as thiol groups of cysteine (e.g., metallothionein; Mercogliano and DeRosier 2006). The most important advantage of metal reducers or chelators is that because of their high electron density, these labels stand out over the average protein density within a cell or organelle. Regular protein domains such as an SH3 domain were shown to be very useful in vitro and on specimens that allowed averaging and statistical difference mapping. However, where protein tags must be visible without computational help, the metal cluster tags are obviously the tags to choose.

The downside of clonable labels in general is that the label may interfere with the function of the labeled protein, or prevents its correct localization in the cell, which has been found in some cases for fluorescent labels such as GFP (Werner et al. 2009). While the first clonable labels we investigated were simply small proteinaceous particles such as an SH3 domain (Wendt et al. 2002; Skiniotis et al. 2003), the more recent ones are either metallothionein (MTH; Mercogliano and DeRosier 2006) or metal-clustering peptides (e.g., the gold-clustering A3-peptide; Slocik et al. 2005). Although there have been a few reports on successful attempts to use MTH as an intracellular label (Diestra et al. 2009a, b; Risco et al. 2012), in our experience, an intracellular application of MTH or the A3 peptide, followed by loading them with gold to generate a visible high-electron density cluster, has proved very challenging. While proteins tagged with a small peptide or a small protein domain may still function properly, once the tag is loaded with metal, there is an increased chance that the physiology of the protein of interest will be affected due to the large mass of the tag. In our own lab, we found almost by accident that MTH clusters zinc very well because we supplemented media with excess zinc (less toxic than gold) to prevent MTH from sequestering essential metals from the cytosol. We succeeded with MTH and A3 peptide applications for in vitro experiments (see Fig. 4; Bouchet-Marquis et al. 2012), but so far we have been able to load intracellular MTH only with zinc. Zinc, however, has a significantly lower electron scattering potential than gold and is easier to detect by electron energy loss spectroscopy (EELS) than gold because unlike gold, zinc produces a sharp energy absorption edge (−18 and −1,020 eV). Nevertheless, EELS is not a current option for vitrified specimens due to the large amount of electron scattering required to generate a useful signal.

## Recent hardware developments for cryo-EM and cryo-ET

The last few years have seen a dramatic change in cryo-EM and cryo-ET data collection through the introduction of the direct electron detector cameras that work with CMOS chips. Similar to regular photography, CMOS chips mark a quantum leap for cryo-EM data acquisition. The direct electron detector technology omits the scintillator and fiber optics, which lowered image quality through their significant point-spread function as well as noise generation by back-scattered electrons. Back-scatter can still be a problem in direct electron detector cameras, but it can be largely overcome by back-thinning the support layer (McMullan et al. 2009b). New high-speed subframe readout procedures allow for some image corrections such as drift already during data acquisition (Jin et al. 2008; Campbell et al. 2012). If the chip is read out fast enough and the rate of incoming electrons is low enough, individual primary electrons can be detected and their exact location estimated, thus eliminating both signal spreading and packet variability as limiting factors (McMullan et al. 2009a). Gatan Inc. has implemented such electron counting in the K2 Summit camera. In addition, the K2 Summit camera has a super-resolution mode, in which the estimates of electron location are used to form an image with pixels half as big as the physical ones. Hence, our expectations from these new cameras are high.

Other significant improvements, especially for cryo-ET, were the development of cartridge-based cryo-stages that disconnected the specimens from the outside. These stages were implemented first in the FEI Polara, and JEOL-3100FCC microscopes, and further perfected with the FEI Titan-Krios microscopes where the cartridge system was combined with an automated multi-specimen autoloader. The significantly better stability of all these stages during tilt-series data acquisition is undisputed. Furthermore, while extremely successful for highest-resolution EM on non-biological material, the benefits of Cs and Cc correctors as well as the constant-current lenses, introduced with the FEI Titan product line, have yet to be carefully evaluated for cryo-ET on biological specimens. But they may be the technologies that will further shape the bright future of cryo-ET on cells and macromolecular structures.
